# Supplementary material for: Disulfiram Protects Against Diet-Induced Obesity by Reprogramming Systemic Lipid Partitioning Independent of GSDMD
Source: bioRxiv. 2026 Feb 9:2026.02.06.704424. Preprint. [Version 1] doi: 10.64898/2026.02.06.704424 (PMC12918825; doi:10.64898/2026.02.06.704424)
Supplement: Supplement 2 [file media-2.pdf]

KEY RESOURCES TABLE

REAGENT or RESOURCE	SOURCE	IDENTIFIER
**Chemicals, Peptides and Recombinant Proteins**		
LPS from E. coli	Enzo Life Sciences	ALX-581-012
Nigericin Sodium Salt	Sigma	N7143
3-(4,5-dimethylthiazol-2-yl)-2,5-diphenyltetrazolium bromide (MTT)	Thermo Fisher	M6494
Pentafluorobenzyl bromide	Sigma	90257
Diisopropylethylamine	Sigma	496219
U-$^{13}C_{16}$ Palmitic acid	Cambridge Isotope Labs	CLM-409-PK
U-$^{13}C_6$ Glucose	Cambridge Isotope Labs	CDLM-3813-PK
U-$^{13}C_6$ Leucine	Cambridge Isotope Labs	CLM-2262-H-PK
D-Glucose	Sigma	G8720
Humulin-R	El Lilly	HI-210
Trizol	Thermo Fisher Scientific	15596018
2ml bead mill tubes	Thermo Fisher Scientific	15-340-153
13x100 mm borosilicate glass tubes	Thermo Fisher Scientific	14-961-27
**Critical Commercial Assays**		
High-Capacity cDNA reverse transcription kit	ABI Biosystems	4368813
TaqMan Fast Advanced Master mix	Thermo Fisher Scientific	4444963
Fast SYBR Green Master Mix	Thermo Fisher Scientific	4385612
Total cholesterol	Infinity	TR13421
Triglycerides	Infinity	TR22421
NEFA	Randox	F115
Insulin	ALPCO	80-INSMSU-E10
Glucagon	Crystal Chem	81518
BCA Protein Assay kit	Thermo Fisher Scientific	23225
**Animals**		
C57BL/6J	JAX Laboratories	000664
C57BL/6J-*Gsdmd*^{em1Vnce}/J	JAX Laboratories	032663
**Diets**		
LFD	Dyets	110700
LFDD	Dyets	103530
HFD	Dyets	101920

HFDD	Dyets	103532
**Software**		
NIH ImageJ	NIH	http://imagej.net
**Equipment/ Instruments**		
QuantStudio 3 Real-time PCR System	Applied Biosystems	https://www.thermofisher.com/order/catalog/product/A28567
Varioskan LUX Multimode Microplate Reader	Thermo Fisher Scientific	https://www.thermofisher.com/us/en/home/life-science/lab-equipment/microplate-instruments/plate-readers/models/varioskan.html
K2 Fluorescent Viability Cell Counter	Nexcelom	https://www.nexcelom.com
ECLIPSE Ti2	Nikon	https://www.microscope.healthcare.nikon.com/products/invertedmicroscopes/eclipse-ti2-series
Element HT5	Heska	https://www.heska.com/product/element-ht5/
Micro- Cell	MicroVet Diagnostics	https://www.microvetdiagnostics.com/Products/Hematology
**Oligonucleotides**		
Antisense oligonucleotides (ASOs)		
Gsdmd	IONIS Pharmaceuticals	
Control	IONIS Pharmaceuticals	
TaqMan probes		
Nlrp3 (Mm00840904_m1)	Applied Biosystems	4331182
Gsdmd (Mm00509958_m1)	Applied Biosystems	4331182
Eukaryotic *18S rRNA* Endogenous Control	Applied Biosystems	4319413E
Quantitative real-time PCR primers	ThermoFisher/IDT	Please see Table S1

Supplemental Table S1: Oligonucleotide sequences for RT-qPCR, related to STAR methods

Oligo Name	Sequence	Source (Ref Seq#, NCBI)
**Oligos from ThermoFisher**		
Mcp1_F	CCTGCTGTTCACAGTTGCC	NM_011333.3
Mcp1_R	ATTGGGATCATCTTGCTGGT	
Ndst1_F	GGCAAGGAGGGCACACGCAT	NM_008306.3
Ndst1_R	GCCGTGCTCGACAGCGAACT	
Cyp2b10_F	GGGAACCTCTTGCAGATG	NM_009999.3
Cyp2b10_R	CCCAGGTGCACTGTGAA	
Cyp27a1_F	GGAGGATTGCAGAACTGGAG	NM_024264.4
Cyp27a1_R	TGCGGGACACAGTCTTTACTT	
Cyp7a1_F	GTCCGGATATTCAAGGATGCA	NM_007824.2
Cyp7a1_R	AGCAACTAAACAACCTGCCAG TACTA	
Ext1_F	GCTCTTGTCTCGCCCTTTTGT	NM_010162.2
Ext1_R	TGGTGCAAGCCATTCCTACC	
Ext2_F	CAGGCAACATATTGAACAGC	NM_010163.2
Ext2_R	CAGGCAACATATTGAACAGC	
**Oligos from IDT**		
Hmgcr_F	GGCCTCCATTGAGATCCG	NM_008255.2
Hmgcr_R	CACAATAACTTCCCAGGGGT	
Fas_F	GGCATCATTGGGCACTCCTT	NM_007988.3
Fas_R	GCTGCAAGCACAGCCTCTCT	
Cd36_F	GCGACATGATTAATGGCACA	NM_001421119.1
Cd36_R	CCTGCAAATGTCAGAGGAAA	
Pparγ_F	GATGCACTGCCTATGAGCAC	NM_011146.4
Pparγ_R	TCTTCCATCACGGAGAGGTC	

Pparα_F	GTCCTCAGTGCTTCCAGAGG	NM_001113418.1
Pparα_R	GGTCACCTACGAGTGGCATT	
Fabp1_F	TCACCATCACCTATGGACCCA	NM_017399.5
Fabp1_R	TCCAGTTCGCACTCCTCCC	
Abcg5_F	GTGCATCTTAGGCAGCTCAG	NM_031884.2
Abcg5_R	TTCACAAACACCTCCCCTTC	
Abcg8_F	CACTGGTCATGGCTGAGAAA	NM_001347418.1
Abcg8_R	TCCGAGGAGAACAAGCTGTC	
Abcg1_F	ATCGAATTCAAGGACCTTTCC	NM_009593.2
Abcg1_R	TTTCCCAGAGATCCCTTTCA	
Scarb1_F	GGCTGCTGTTTGCTGCG	NM_001424495.1
Scarb1_R	GCTGCTTGATGAGGGAGGG	
Apoe_F	AACCGCTTCTGGGATTACCT	NM_009696.4
Apoe_R	CAGTGCCGTCAGTTCTTGTG	
Ldlr_F	GCATCAGCTTGGACAAGGTGT	NM_001252659.1
Ldlr_R	GGGAACAGCCACCATTGTTG	
Lrp1_F	CCTGAAGGGCTTTGTGGAT	NM_008512.2
Lrp1_R	TAGAAGTTTCCCGTCAGCCA	
Pgc1α_F	AACCACACCCACAGGATCAGA	NM_001402988.1
Pgc1α_R	TCTTCGCTTTATTGCTCCATGA	
Nr1h3_F	GCTCTGCTCATTGCCATCAG	NM_001177730.1
Nr1h3_R	TGTTGCAGCCTCTCTACTTGGA	
