# Supplementary figures and images for: Disulfiram Protects Against Diet-Induced Obesity by Reprogramming Systemic Lipid Partitioning Independent of GSDMD
Source: bioRxiv. 2026 Feb 9:2026.02.06.704424. Preprint. [Version 1] doi: 10.64898/2026.02.06.704424 (PMC12918825; doi:10.64898/2026.02.06.704424)

**A**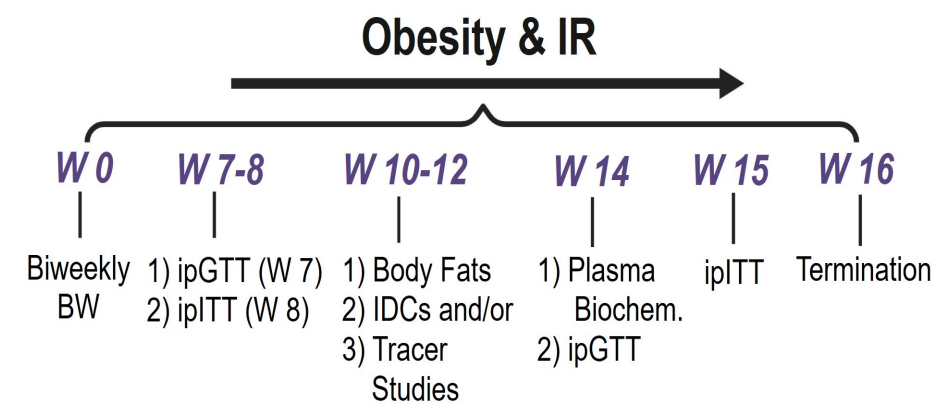**B**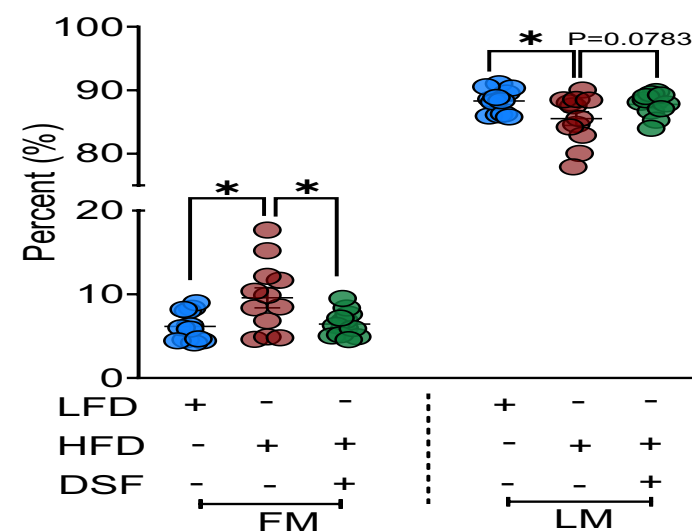**C**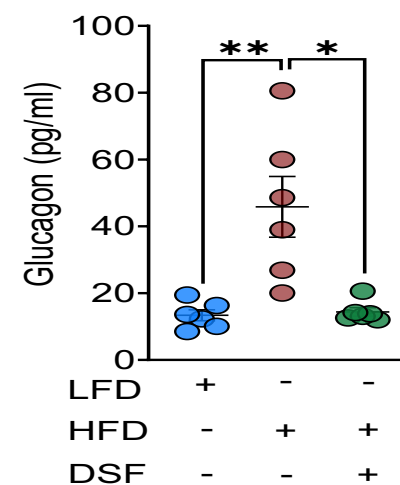**D**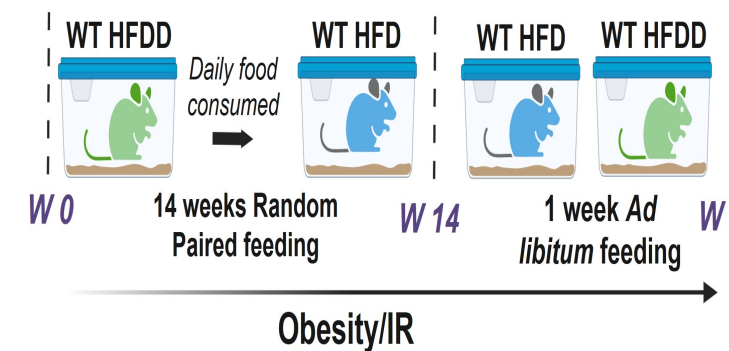**E**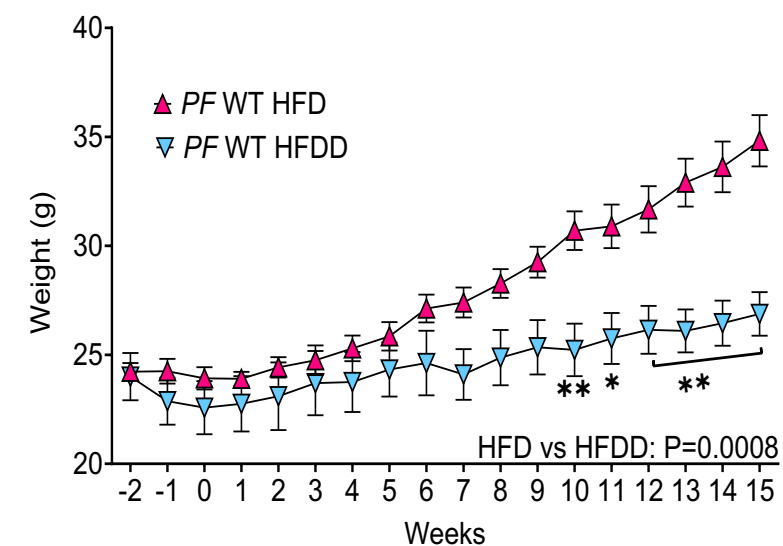**F**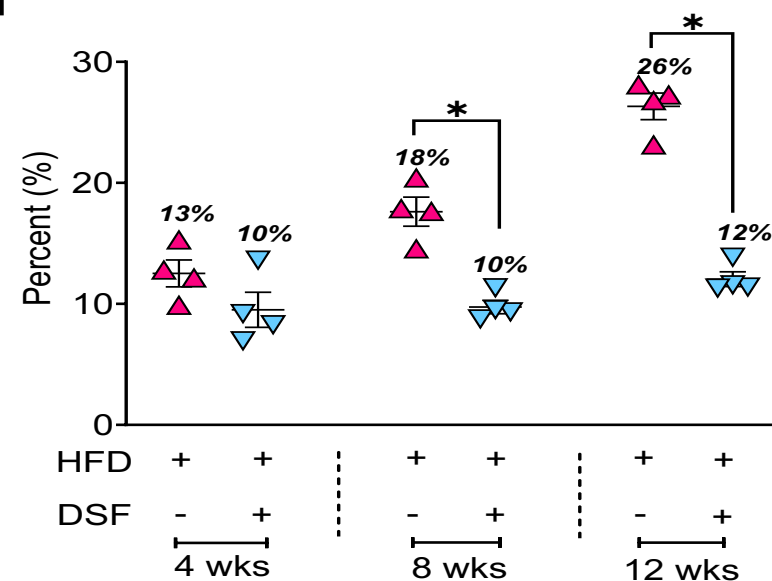**G**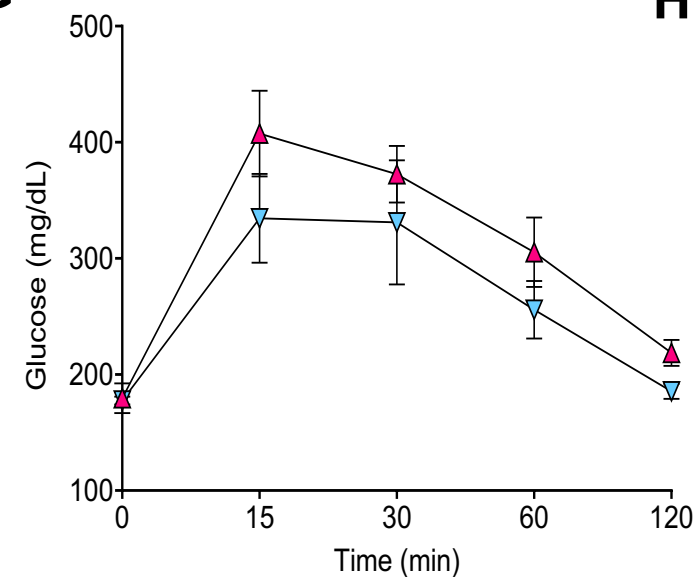**H**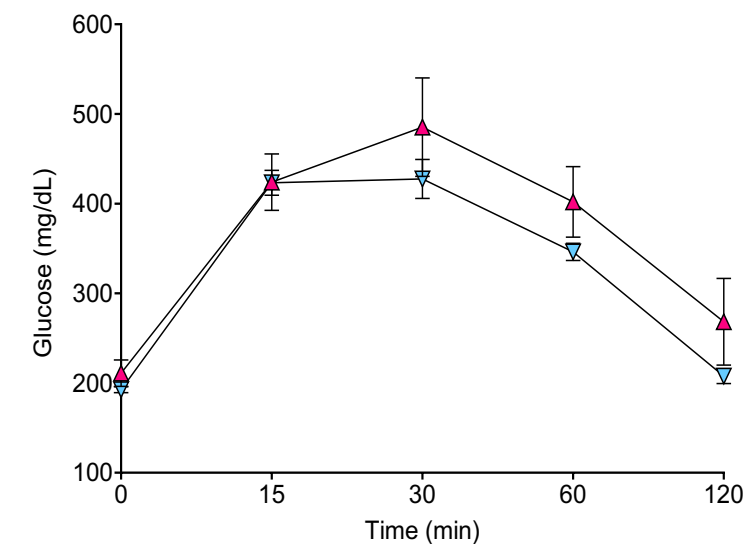**I**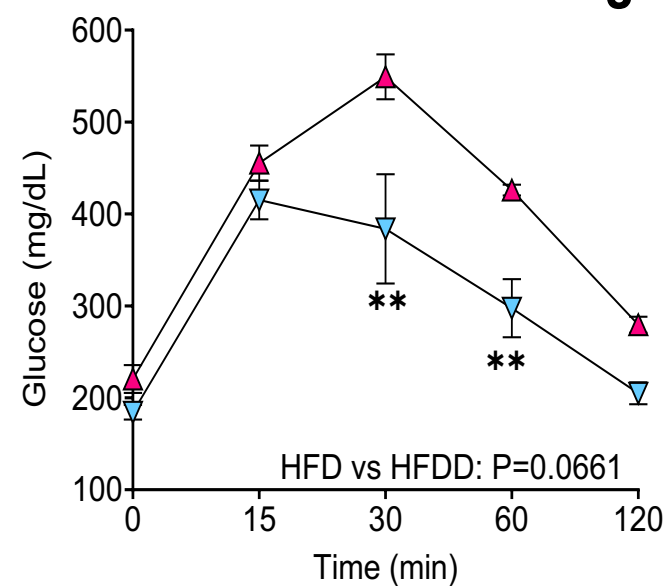**J**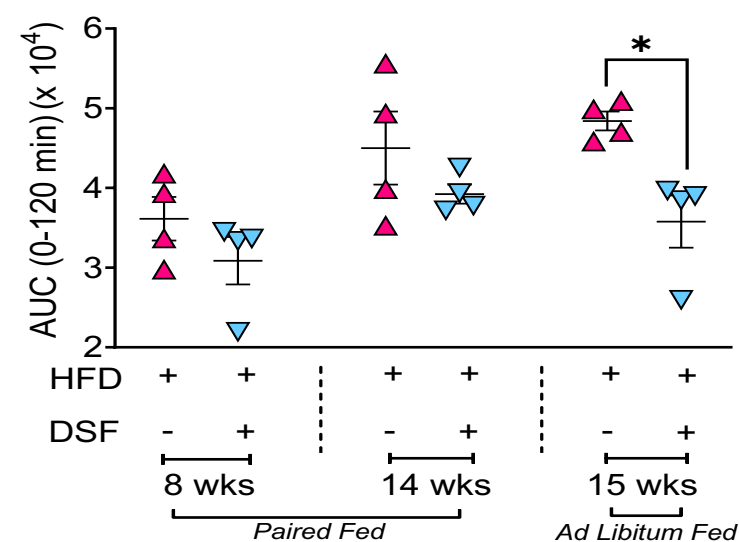**K**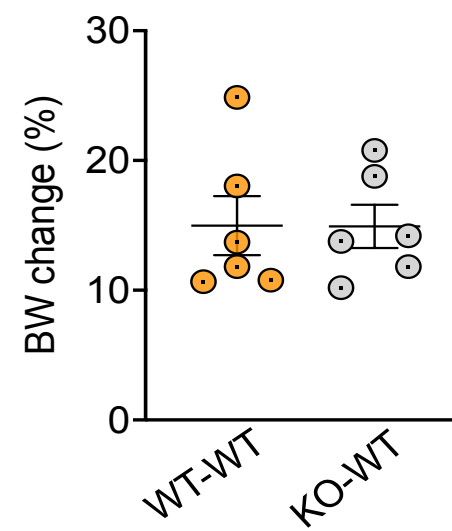**L**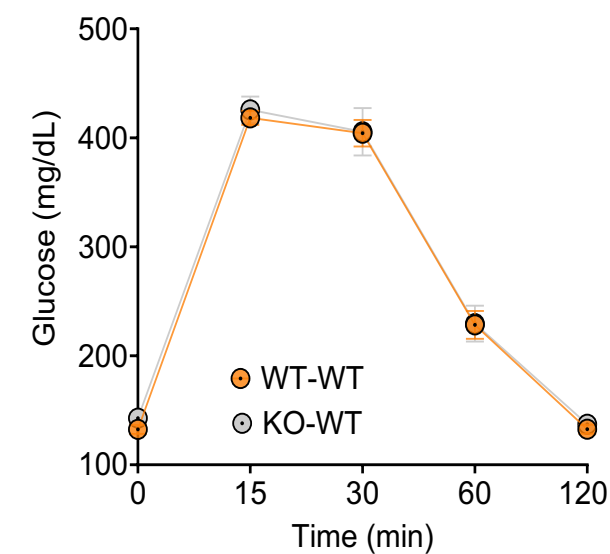**M**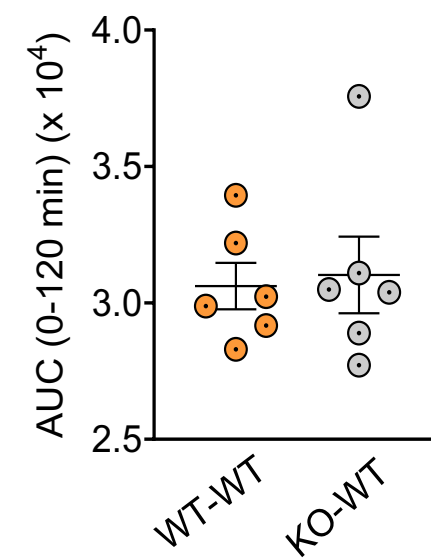

Supplement: Supplement 3 [file media-3.pdf]

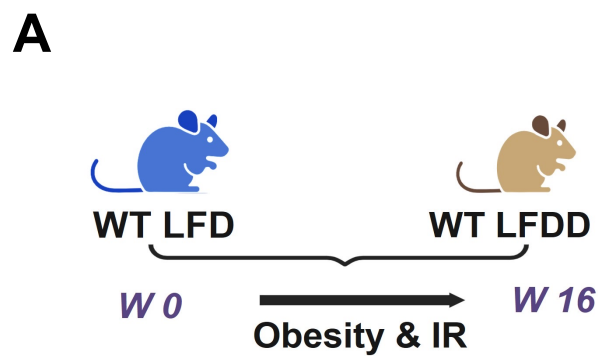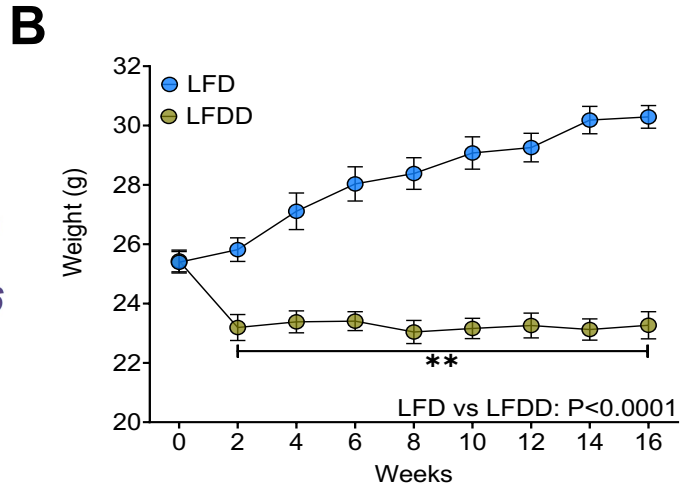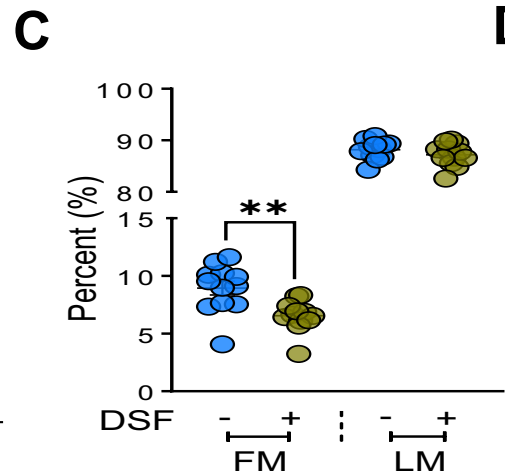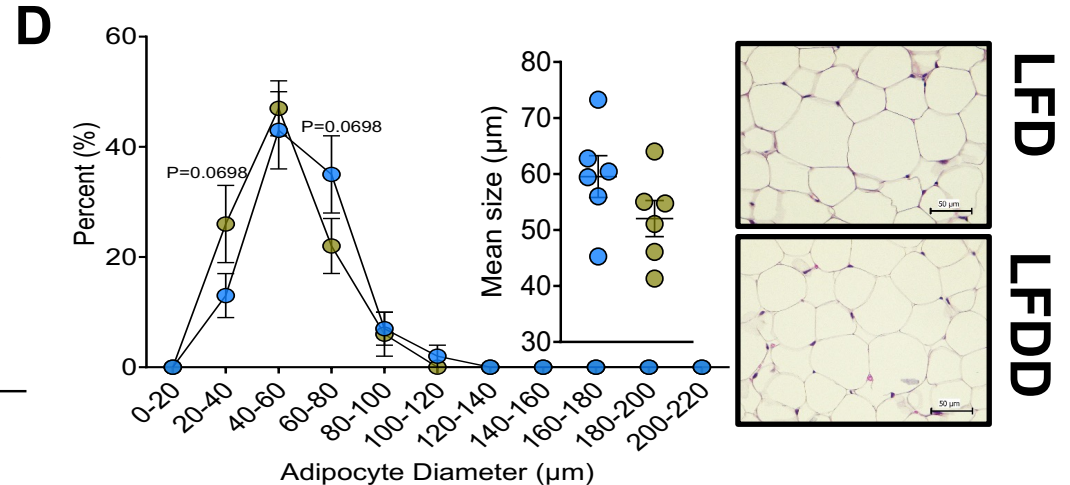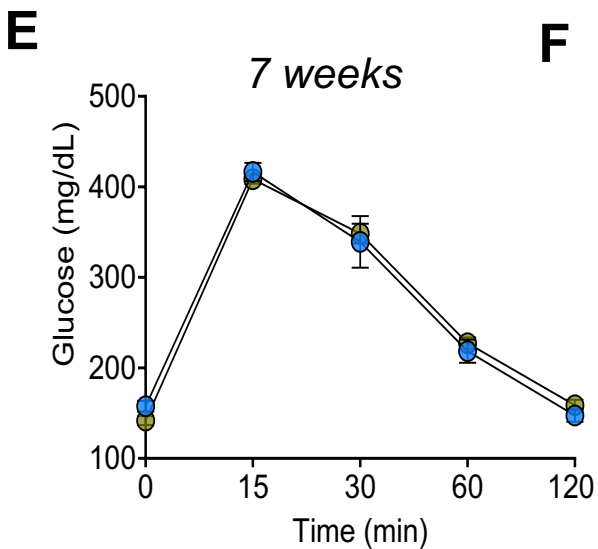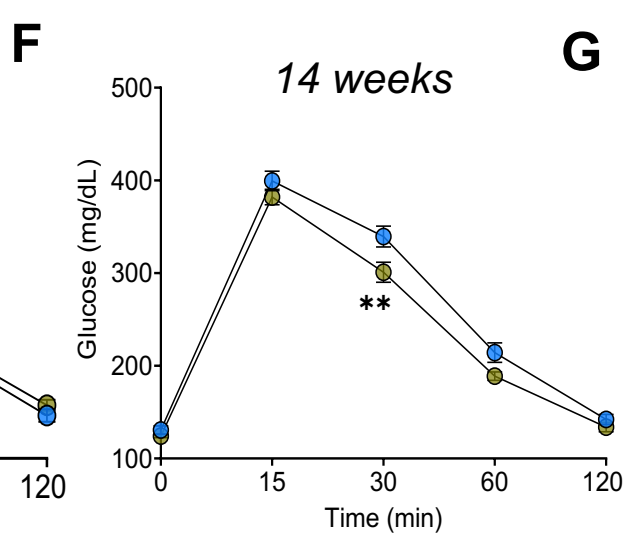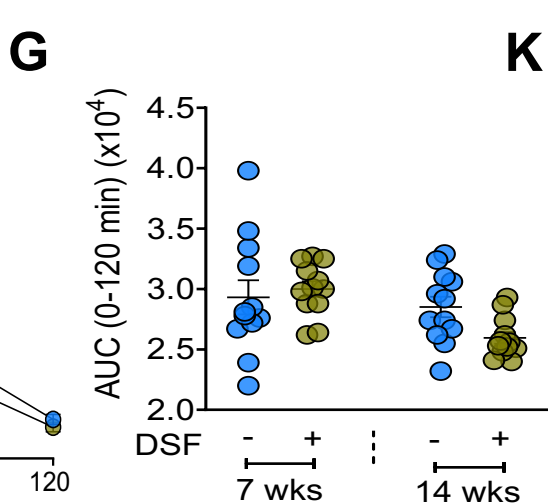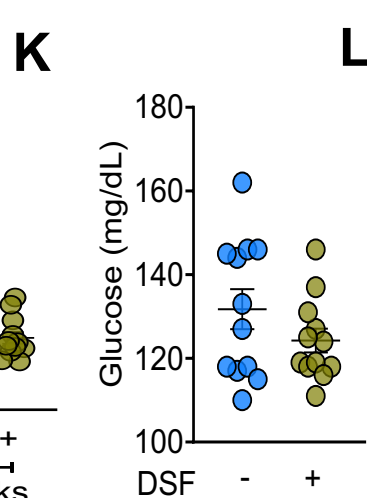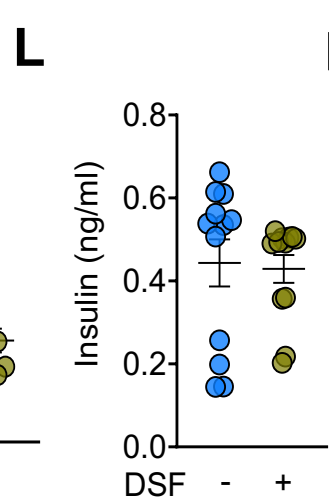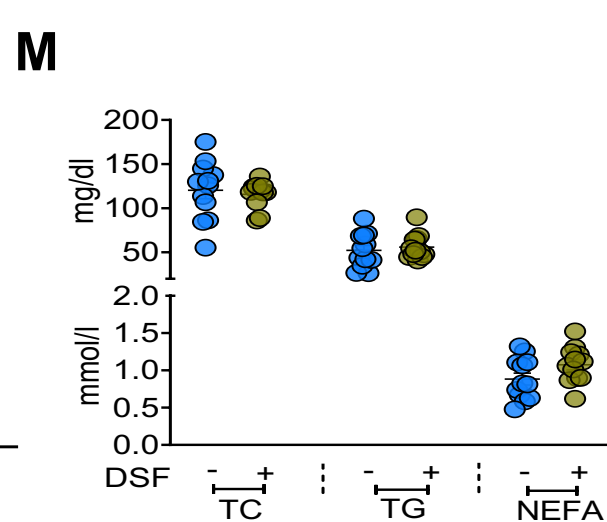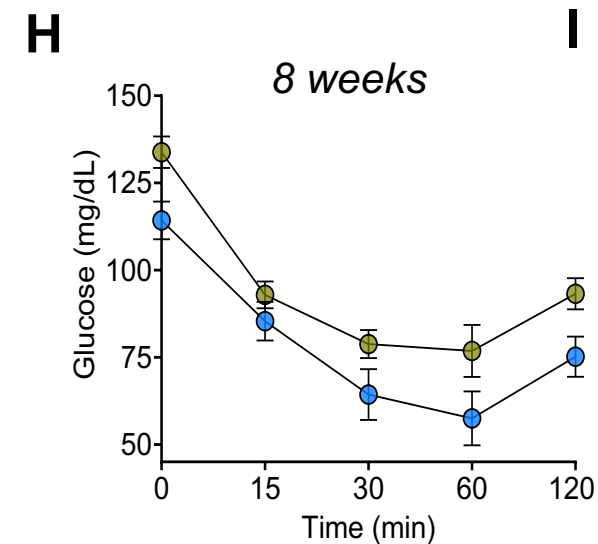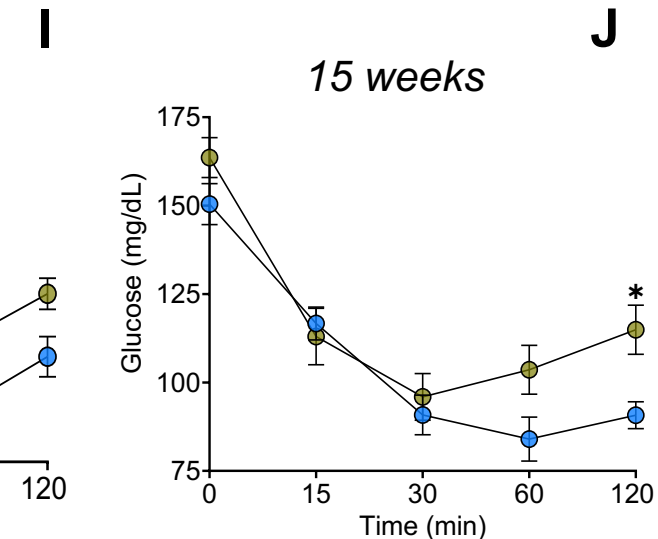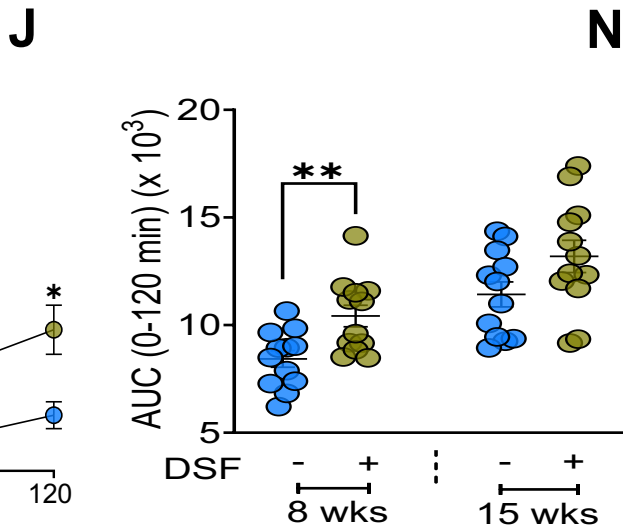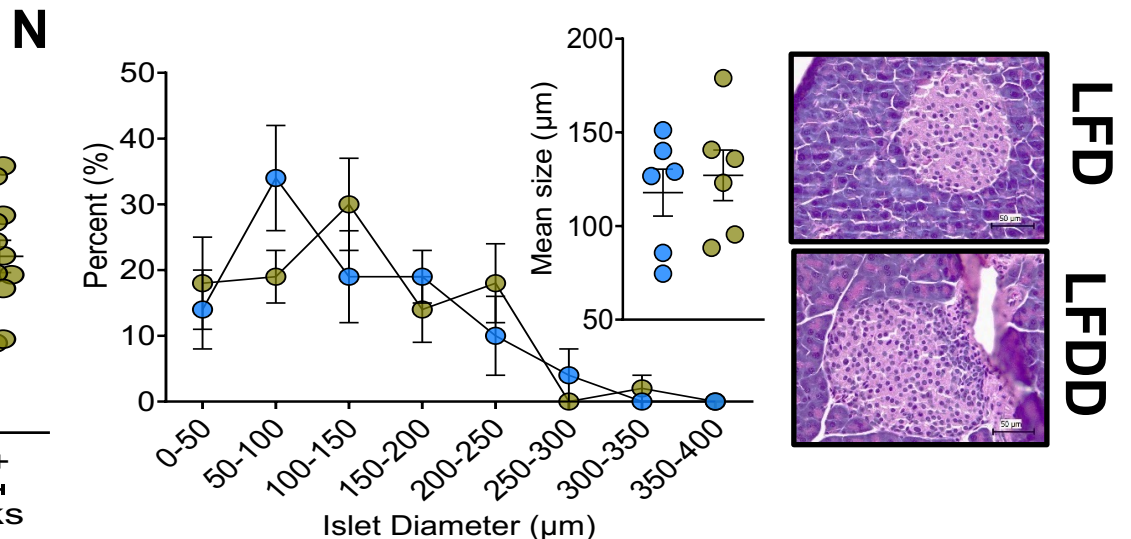

Supplement: Supplement 4 [file media-4.pdf]

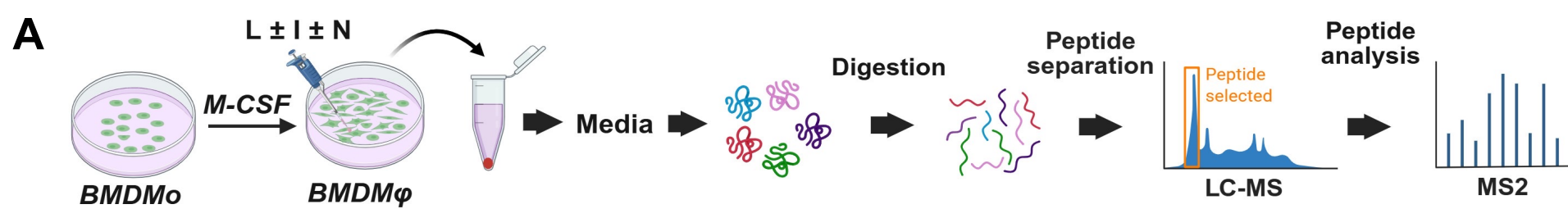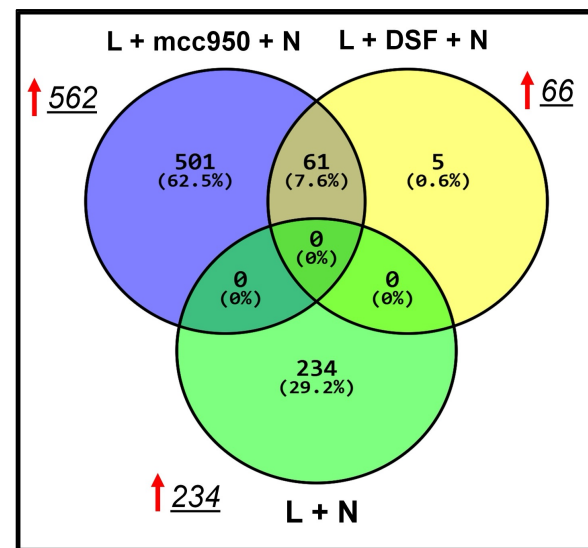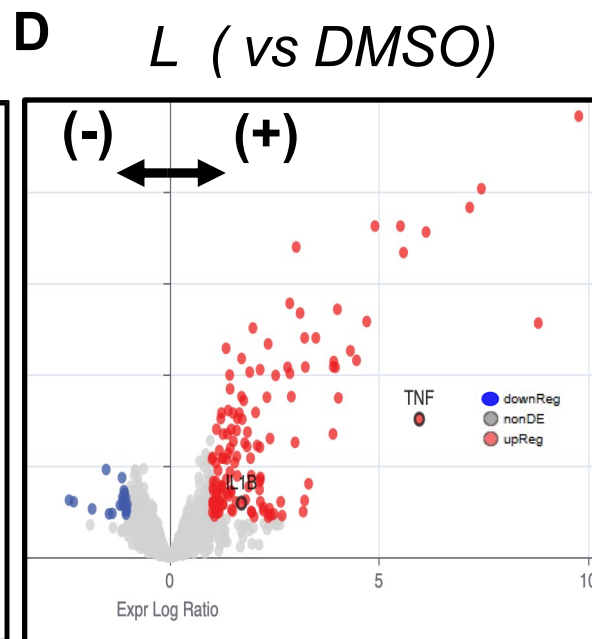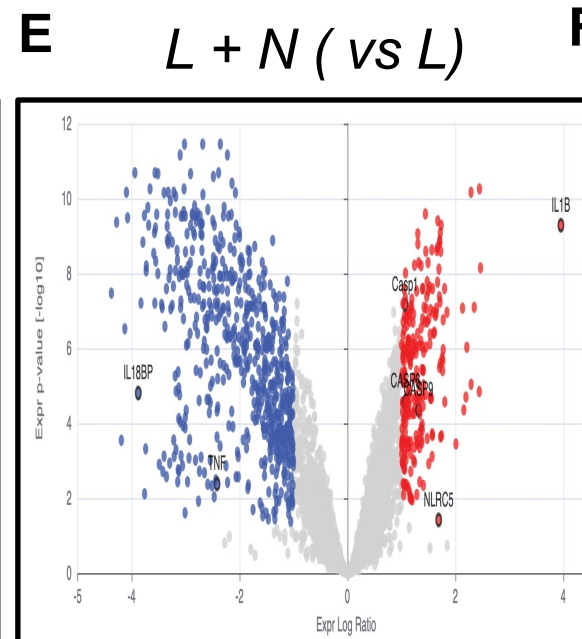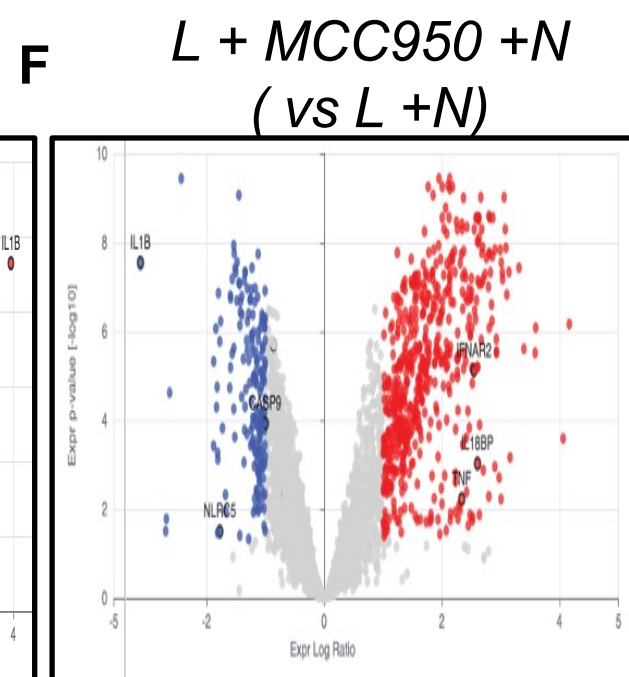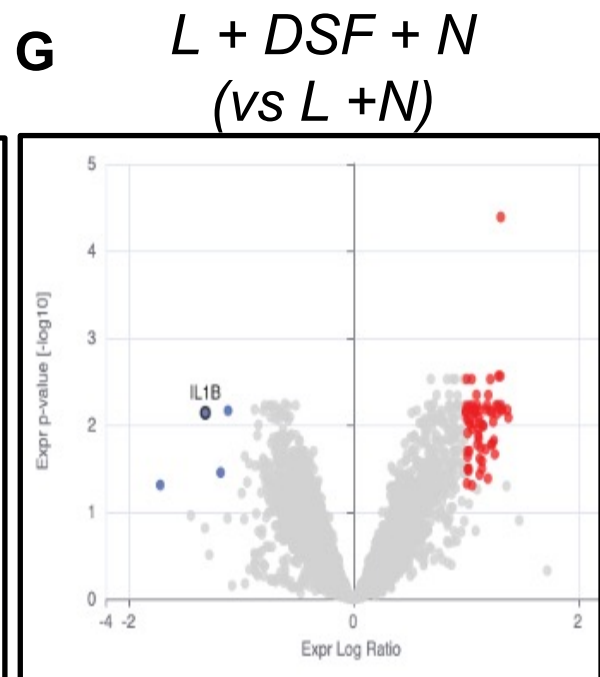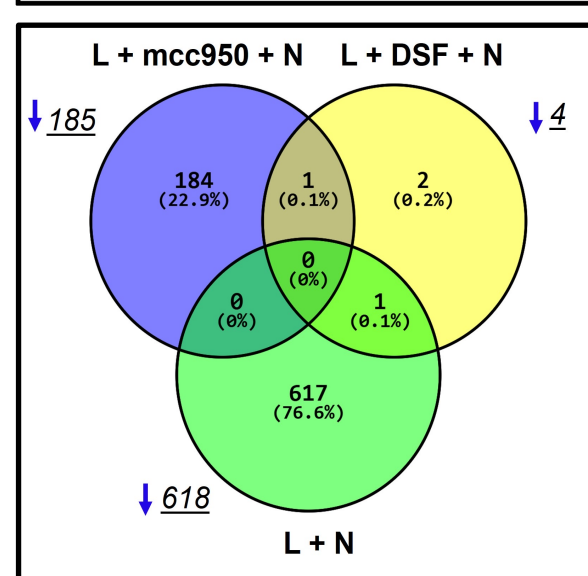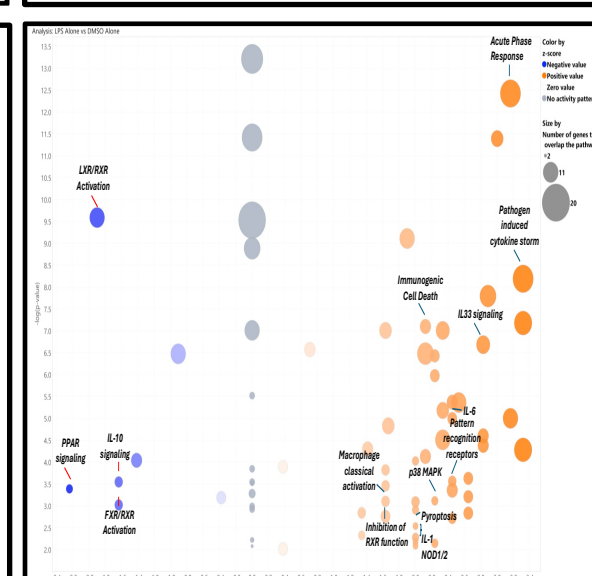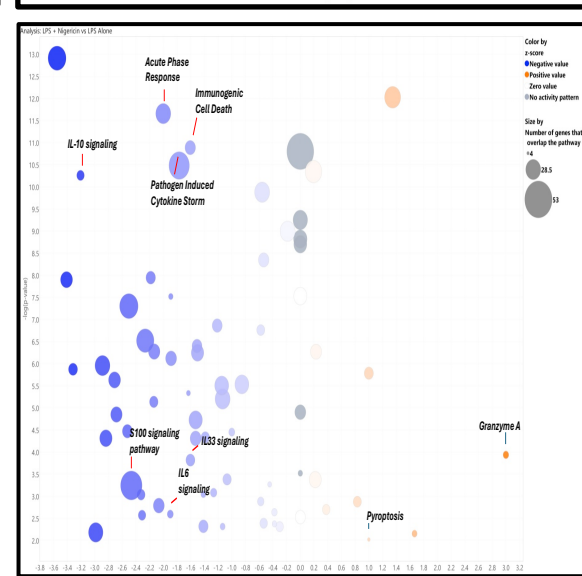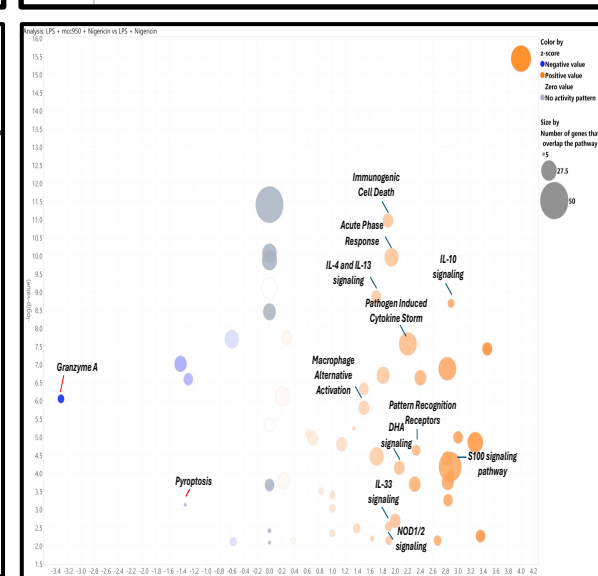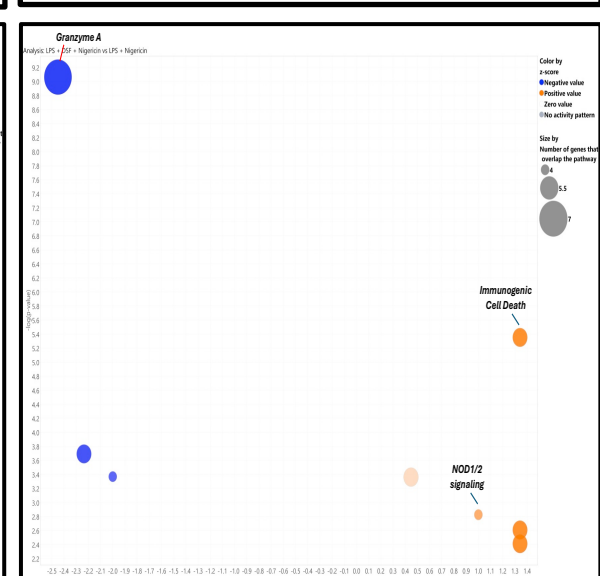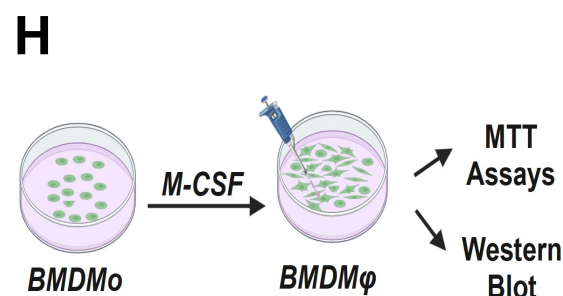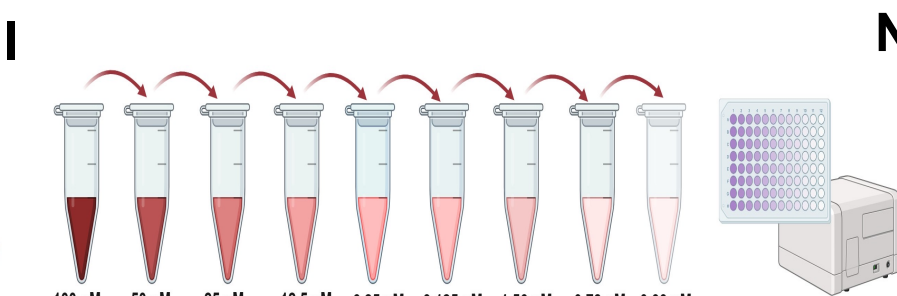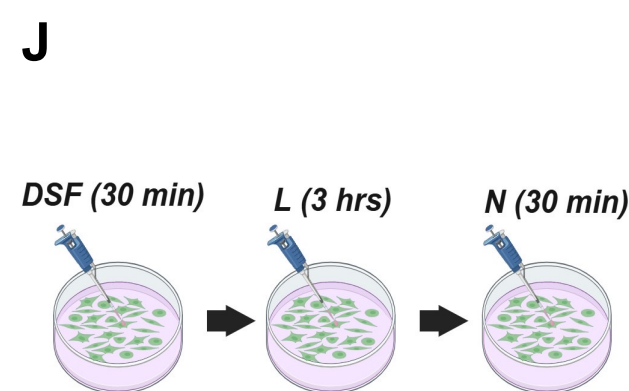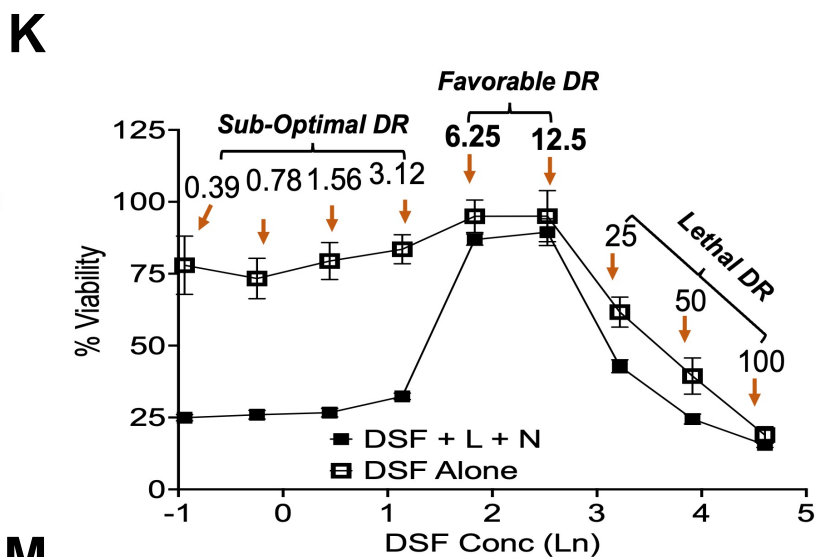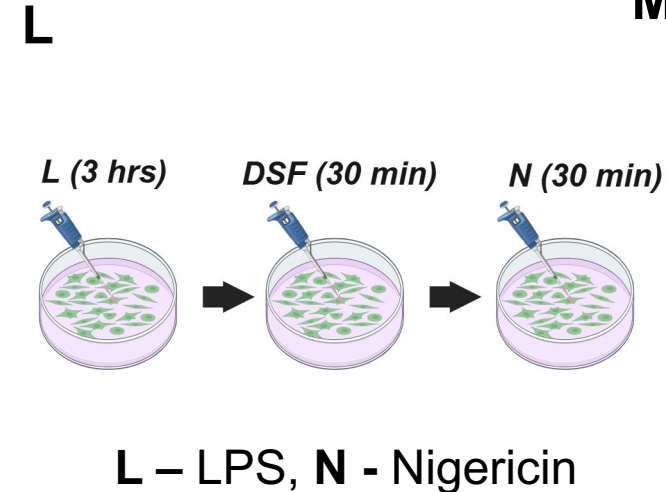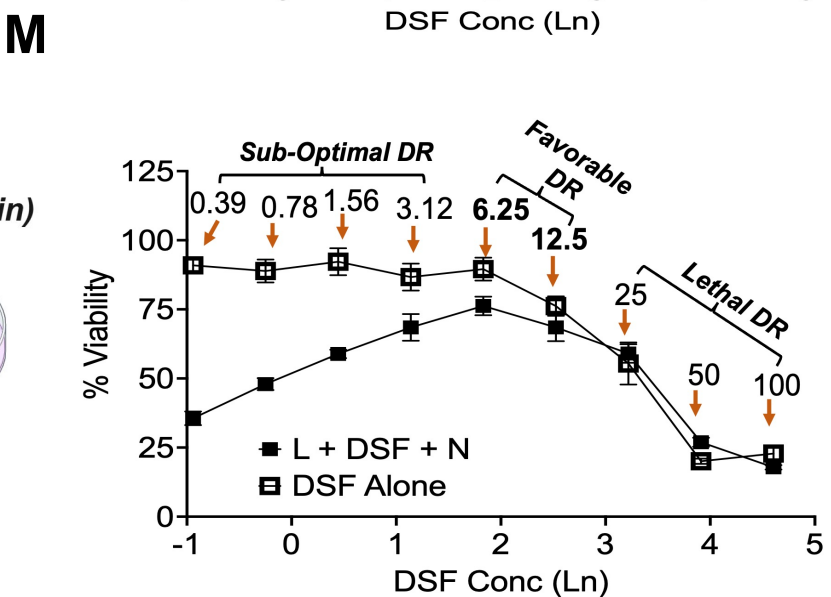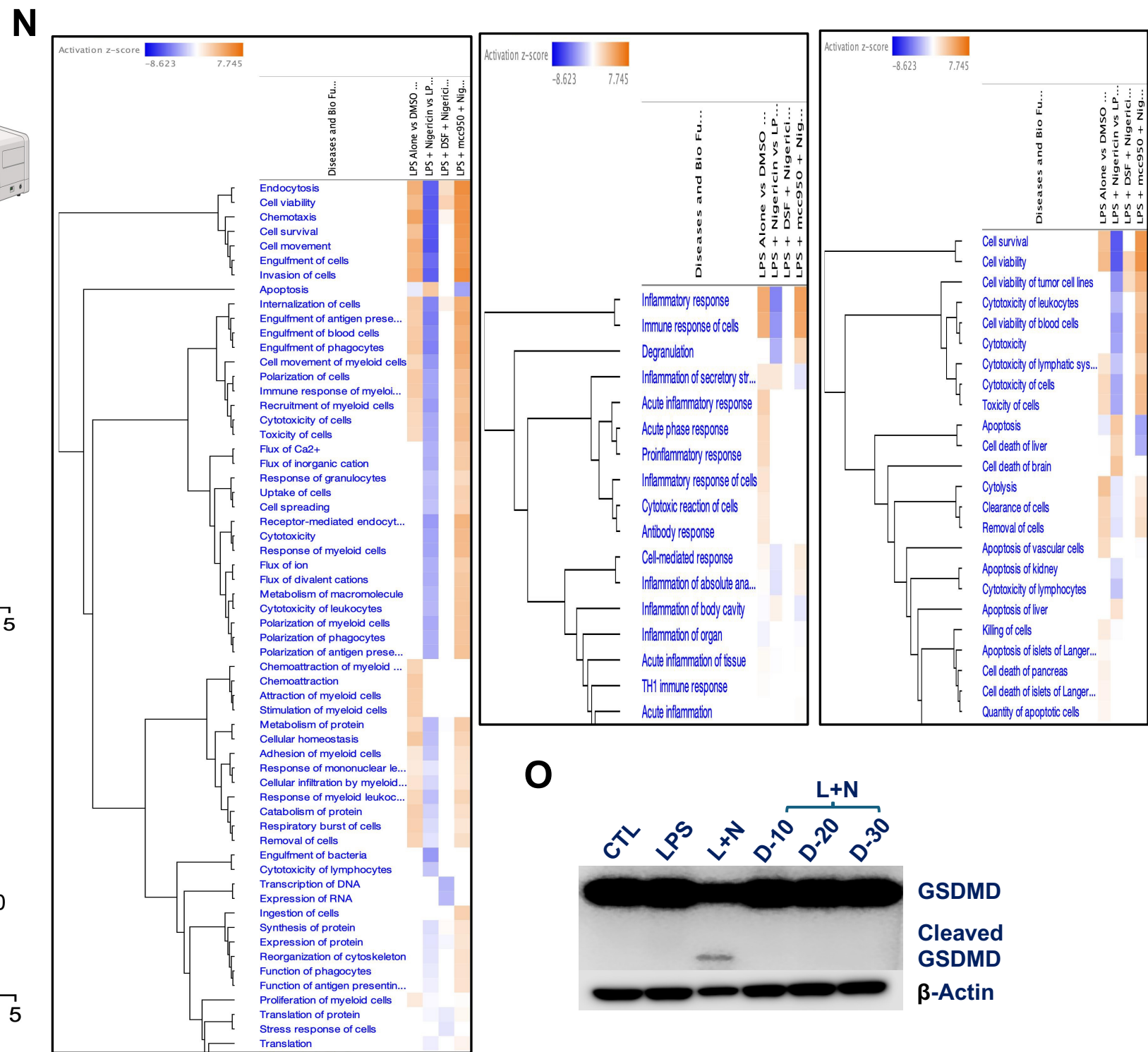

Supplement: Supplement 5 [file media-5.pdf]

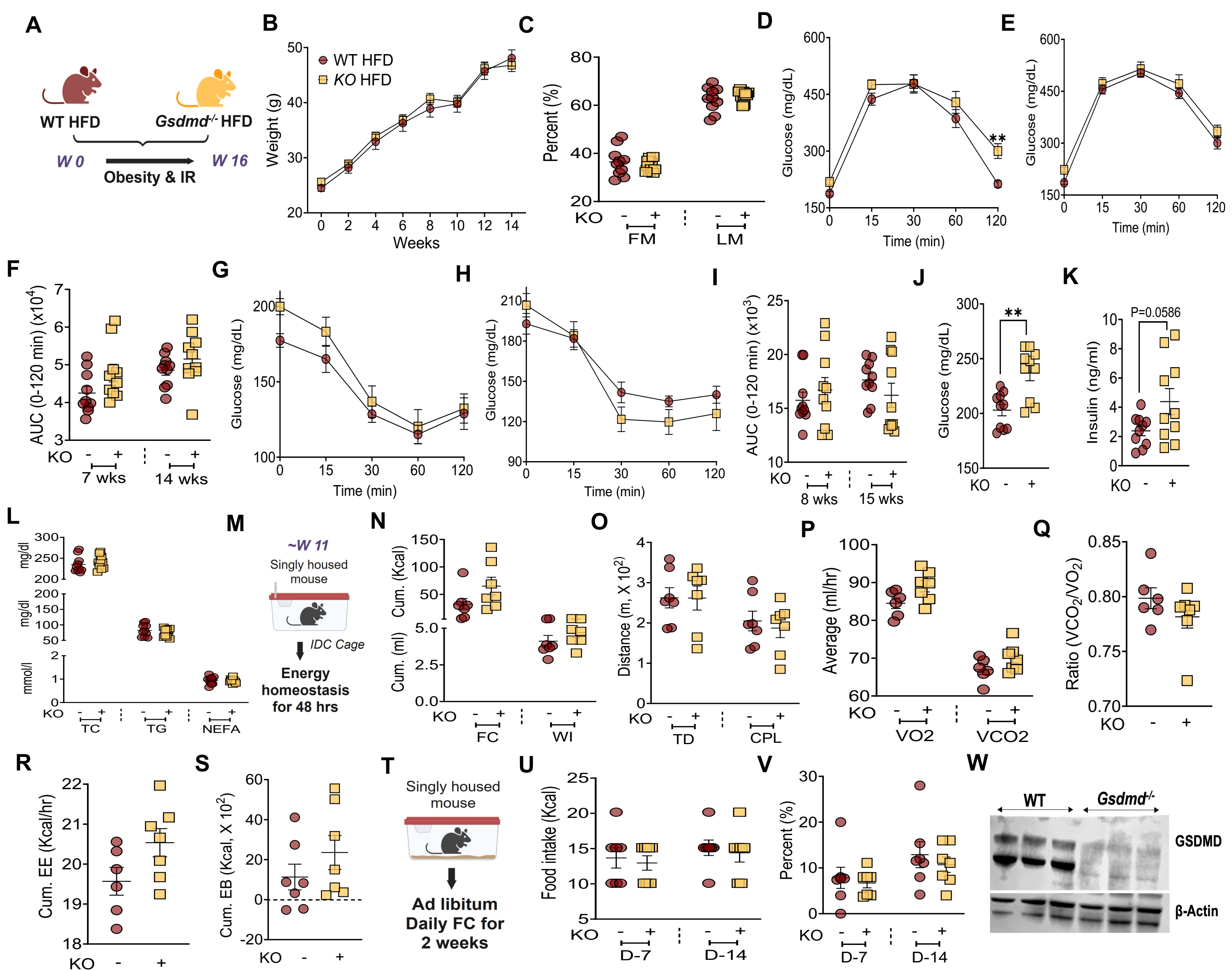

Supplement: Supplement 6 [file media-6.pdf]

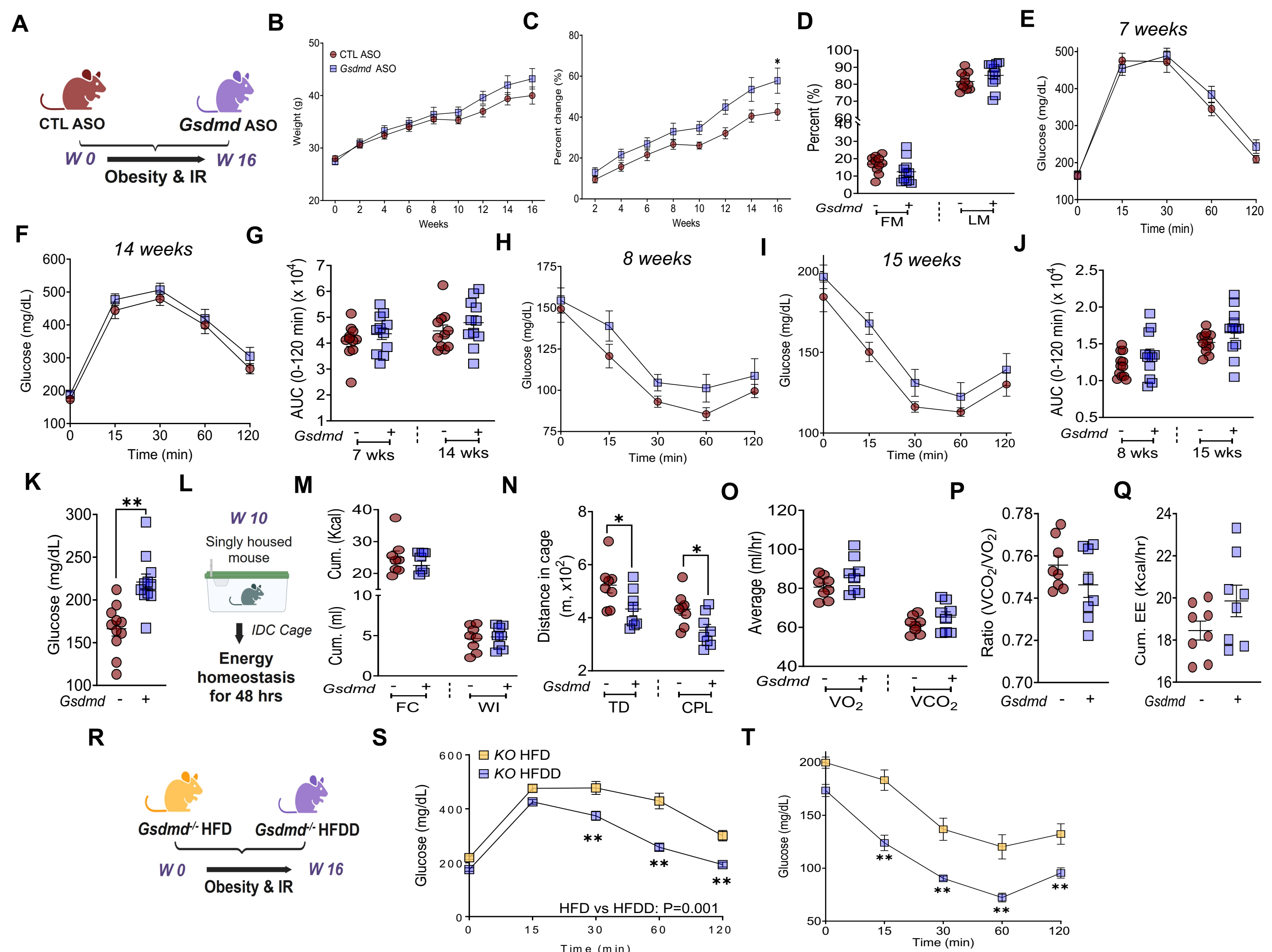

Supplement: Supplement 8 [file media-8.pdf]

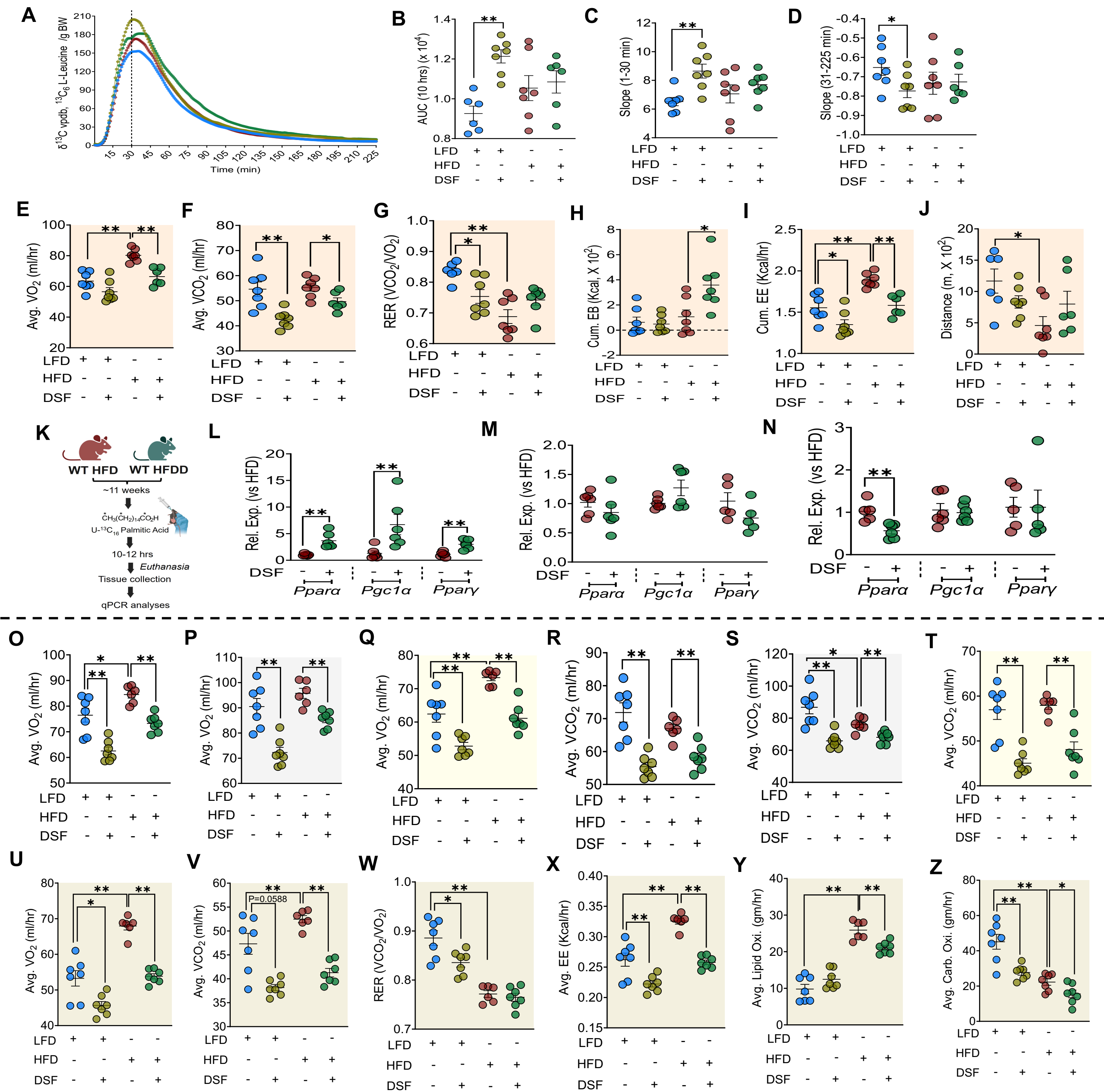

Supplement: Supplement 9 [file media-9.pdf]
