# Supplementary material for: Disulfiram Protects Against Diet-Induced Obesity by Reprogramming Systemic Lipid Partitioning Independent of GSDMD
Source: bioRxiv. 2026 Feb 9:2026.02.06.704424. Preprint. [Version 1] doi: 10.64898/2026.02.06.704424 (PMC12918825; doi:10.64898/2026.02.06.704424)
Supplement: Supplement 7 [file media-7.pdf]

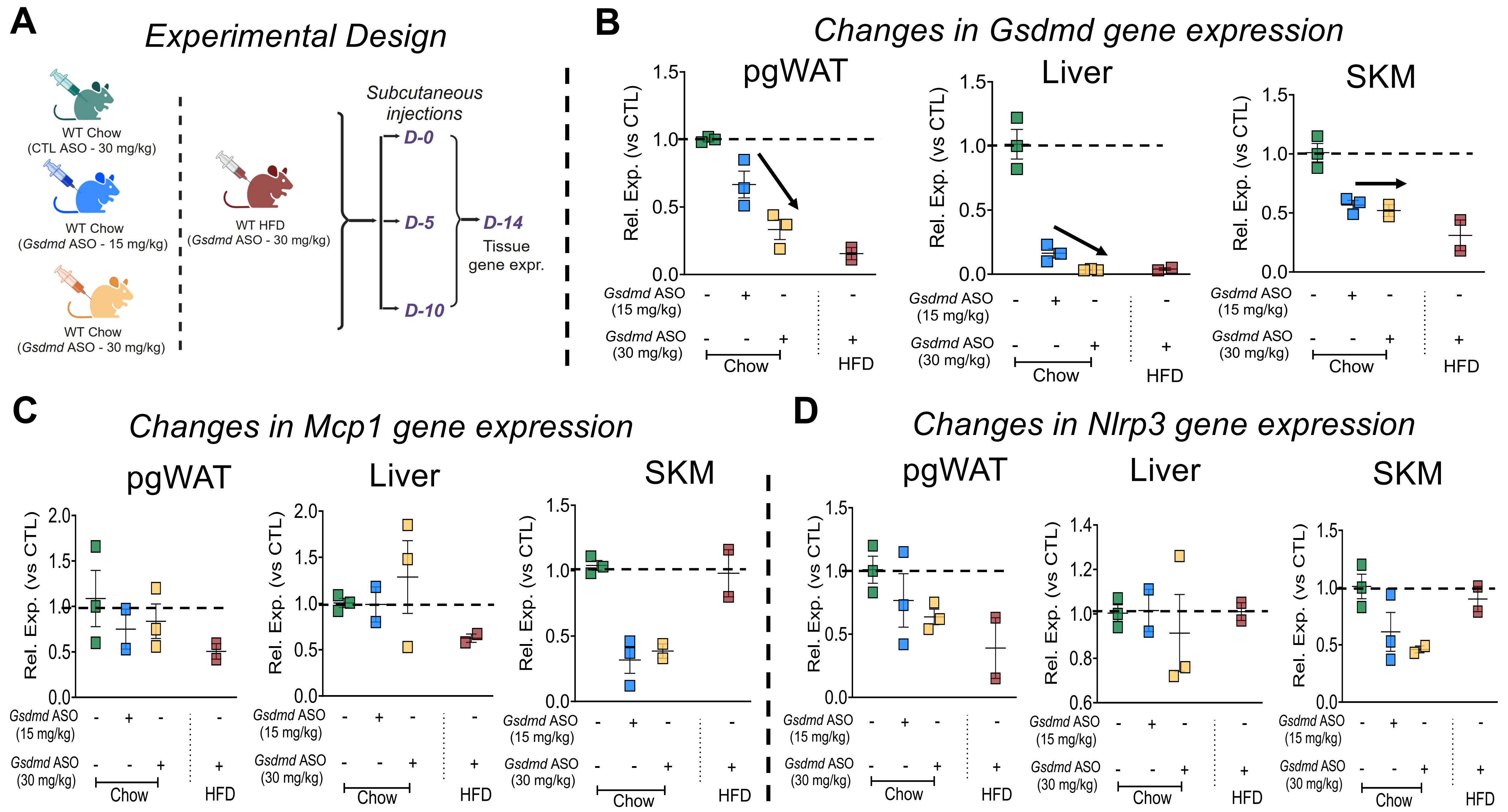
A
Experimental Design
WT Chow
(CTL ASO - 30 mg/kg)
WT Chow
(Gsdmd ASO - 15 mg/kg)
WT Chow
(Gsdmd ASO - 30 mg/kg)
WT HFD
(Gsdmd ASO - 30 mg/kg)
Subcutaneous injections
D-0
D-5
D-10
D-14
Tissue gene expr.
B
Changes in Gsdmd gene expression
pgWAT
Liver
SKM
Rel. Exp. (vs CTL)
Gsdmd ASO (15 mg/kg)
Gsdmd ASO (30 mg/kg)
Chow
HFD
C
Changes in Mcp1 gene expression
pgWAT
Liver
SKM
D
Changes in Nlrp3 gene expression
pgWAT
Liver
SKM
